# The Role of miRNA in Ovarian Cancer: an Overview

**DOI:** 10.1007/s43032-021-00717-w

**Published:** 2022-01-01

**Authors:** Lihui Zhao, Xiaolei Liang, Liyan Wang, Xuehong Zhang

**Affiliations:** Key Laboratory for Reproductive Medicine and Embryo of Gansu, First Affiliated Hospital, Lanzhou University, No.1, Donggangxi Rd, Chengguan District, Lanzhou City, Gansu 730000 People’s Republic of China

**Keywords:** Ovarian cancer, miRNA, Treatment, Prognosis

## Abstract

Ovarian cancer (OC) is a highly malignant disease that seriously threatens women’s health and poses challenges for clinicians. MicroRNAs (miRNAs) have recently been intensively studied in the field of oncology due to their regulatory roles in gene expressions through RNA degradation and/or translation inhibition. This review summarizes the current studies on miRNAs in OC and introduces the latest updates of miRNAs in the early screening, treatment, and prognostic prediction of OC, thereby demonstrating the clinical significance of miRNAs in OC. Further exploration on potential targets of miRNAs in OC may provide new insights on optimizing the diagnosis and treatment of OC. MiRNAs are important driving factors for the progression of OC and the dysregulation of miRNAs can serve as biomarkers in the diagnosis, treatment and prognosis of OC. Therefore, miRNAs are potential biological targets for early screening, targeted therapy, drug resistance monitoring, and prognosis improvement in malignancies such as OC.

## Introduction

Ovarian cancer (OC) is a highly malignant entity that affects women in different age groups. The 2020 data shows that there are approximately 313,959 new cases of OC worldwide, of which approximately 207,252 died [http://globocan.iarc.fr]. It is the seventh most common cancer and eighth leading cause of death in women with cancer in a global scale. Thus, OC is a global health burden that calls urgently for studies providing new solutions to improving disease prognosis. Main histological types of OCs include epithelial tumors, germ cell tumors, sex cord-stromal cell tumors, and metastatic tumors, among which epithelial type is the most common one, accounting for about 50–70% of all OCs. Age, family history, and genetic alteration (such as BRCA1/2) are important risk factors for the pathogenesis of OC. At early stage, patients with OC are typically asymptomatic. Meanwhile, some patients may present with non-specific symptoms that are difficult to be differentiated with those with the digestive and urinary systems. Therefore, early identification of OCs is difficult based on routine diagnostic and treatment procedures, gynecological examination, serum CA125, and transvaginal ultrasound examination [1]. Consequently, more than half of patients with OC are diagnosed at advanced stages [2], leading to an unsatisfactory 5-year survival rate of only 20–25% [3, 4]. Contrarily, in patients who were diagnosed at early stages, the 5-year survival rate could reach 90% [5]. The current standard of care for OC is a comprehensive treatment scheme with tumor cytoreductive surgery plus chemotherapeutic regimen such as platinum-based agents. Although the complete remission rate was reported to be as high as 60 to 80%, nearly 50% of patients will develop chemotherapy resistance or relapse in the later stage [6], which poses further challenges in the treatment of OC. Therefore, identification of an effective target for early screening, treatment, and prognostic prediction of epithelial ovarian cancer is necessary for researches in this field.

MicroRNAs (miRNAs) are a class of endogenous non-coding RNAs found in eukaryotes with regulatory functions and normally about 20 to 25 nucleotides in length, which can be isolated from tissues, cells, and body fluids (such as blood and urine, etc.). The main function of miRNAs is to regulate gene expressions in organisms through degradation or translation inhibition of target mRNAs [7]. A single miRNA can target and regulate hundreds of mRNAs by affecting the expression of multiple genes when they interact with targeted mRNAs. Studies have shown that miRNAs are involved in the development and progression of many diseases [8]. Moreover, miRNAs play important roles in various biological processes related with cancer, such as tumorigenesis, cell proliferation, differentiation, apoptosis, angiogenesis, invasion and metastasis, tumor resistance, epithelial-mesenchymal transition, and prognosis. During the pathogenesis of cancers, miRNAs may act as a tumor factors or tumor suppressors [9]. Therefore, potential roles of miRNAs in development and progression of OCs are as of great significance in the field of oncology. This review introduces the latest study results of miRNA in OC in detail, providing insights for subsequent in-depth explorations.

## History and Function of miRNAs

**Fig. 1:**
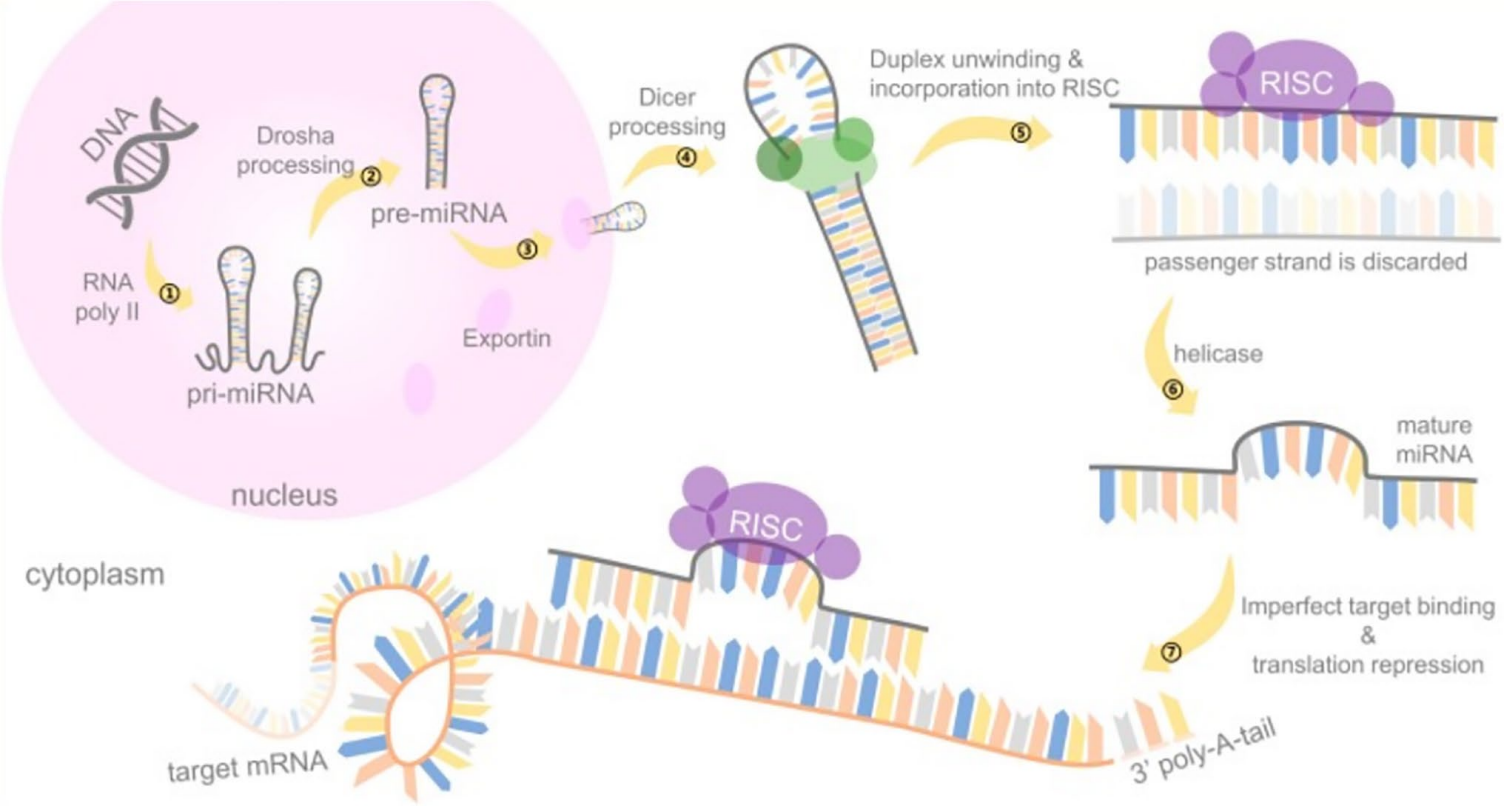
Schematic illustration of miRNA synthesis process

The miRNA is a class of endogenous non-coding RNAs with various regulatory functions, which was first discovered in nematodes in 1993 by Lee et al. [10]. Originally, miRNA is transcribed into long double-stranded precursors by RNA polymerase II in the nucleus [11] and split into 60-to-75-nucleotide hairpin stem loop structure by Drosha (endonuclease III), also known as precursor RNA [12]. The precursor RNA is transported into the cytoplasm through the Expotin 5 protein and processed by the cleavage enzyme to form a mature miRNA, which is then combined with the AGO protein to form the RNA silencing complex (RISC) [13]. The RISC and the specific binding site of the target gene mRNA 3'UTR region regulate the target gene by directly degrading the mRNA or inhibiting protein synthesis [14]. Target gene downregulation by miRNAs mainly depends on the degree of complementarity with the 3’ region of the target gene. MiRNAs that are completely or largely complementary to the region can cleave the target gene, while the lower degree of complementarity can induce silencing and subsequent downregulation of the target gene [15]. Studies have demonstrated that miRNAs participate in a variety of biological activities through negative regulation of target genes, and thereby regulate tumorigenesis, proliferation, invasion, and metastasis of cancers [16]. The schematic illustration of the miRNA synthesis process is shown in Fig. 1 [17].

## Preliminary Studies on miRNAs

MiRNAs secreted by specific cells were found in extracellular vesicles in body fluids, which were first discovered in serum by Han et al. [18], and subsequently separated in urine, saliva, and other body fluids. Further research indicated that miRNAs in body fluids are stable and independent of internal or external factors, and that distinct miRNA expression patterns were present under different pathophysiological conditions [19]. Multiple miRNAs were found to be up- or downregulated in the cervical vaginal fluid of patients with HPV16 infection [20–22]. Thus, potential markers targeting the regulation of miRNA-induced cervical cancer after HPV16 infection can be further identified through bioinformatic analysis. Prostate-specific antigen (PSA) is a biomarker for prostate cancer, and studies have found that certain serum miRNAs can better distinguish benign prostate hyperplasia from prostate cancer [23, 24] comparing to PSA. Moreover, various differentially expressed miRNAs have also been identified in other malignant diseases such as gastric cancer and lung cancer [25, 26]. Due to their preferable features like stable expression and high specificity, miRNAs in body fluids are expected to work as ideal biomarkers and novel targets for the diagnosis and treatment for malignant tumors.

## miRNA Expression in Ovarian Cancer

As a regulating factor of gene expression, miRNAs are either upregulated or downregulated in various malignancies, thus functioning as oncogenes or suppressor genes. Misexpression of miRNAs can affect its targeted genes and thereby lead to the initiation of oncogenesis owing to mechanisms including genetic alterations (such as chromosome rearrangements, deletions, and mutations), epigenetic changes, and abnormalities in transcription and post-transcriptional control [27]. Similarly, miRNAs also play important roles in tumorigenesis and progression of OC, which can serve as detectable serum markers. The diagnostic value analysis of miRNAs for OC exhibited a combined sensitivity of 0.75 and a specificity of 0.75, with an AUC of 0.82 [28]. In addition, the latest research found that compared with traditional miRNA detection, graphene oxide (GO)-based qRT-PCR detection can significantly improve the specificity and sensitivity of miRNA detection (sensitivity, 0.91; specificity, 1.00) [29]. Evidence has demonstrated that circulatory miRNAs can potentially serve as non-invasive biomarker for OC. Several cohort studies using PCR analysis revealed a variety of up- and downregulated miRNAs in OC. Meanwhile, the role of miRNAs in tumorigenicity was further confirmed in ovarian cancer cells and animal experiments [8, 30, 31]. This indicates the possibility to utilize the differential expression profile of miRNAs in OC to effectively distinguish benign ovarian tumors from ovarian cancer, so as to facilitate early detections of OC. Due to their effects on cell proliferation, miRNAs can also work as therapeutic targets for the treatment of OC. Differential expressions of miRNAs in OC is shown in Table 1.

**Table 1:** miRNA expression profile in ovarian cancer

Sample	Upregulated	Downregulated	References
formalin-fixed paraffin-embedded (FFPE) samples	miR-93, miR-325, miR-200a, miR-200b, miR-200c, miR-141, miR-429, miR-492, miR-182	miR-145, miR-152, miR-214, let-7a, let-7b, let-7c	Braicu et al. [30]
ascitic fluid	miR-200a, miR-200b, miR-200c, miR-182, miR-203a, miR-135b	miR-451a	Záveský et al. [31]
cancer associated fibroblasts (CAFs)	miR-155	miR-214, miR-31, miR-199a, let-7b	Mitra et al. [32]
plasma	miR-200c, miR-221, miR-21-5p, miR-484	miR-195, miR-451	Oliveira et al. [33]
serum	miR-99a, miR-100, miR-125b, miR-139, miR-451, miR-500a, miR-1290, miR-3131, miR-3153		Yoshimura et al. [34]
serum	miR-30a, miR-26b, miR-628, miR-520c, miR-486	miR-596	Sommerov et al. [35]
ovarian cancer tissue	miR-182, miR-183, miR-96, miR-182, miR-141, miR-15b, miR-130b, miR-135b	miR-1271, miR-574	Wang et al. [36]
ovarian cancer sample	miR-224-5p, miR-200a	miR-99a-5p, miR-320a, miR-150, miR-30d, miR-342, miR-424, miR-502	Krasniqi et al. [37]

## The Significance of miRNA in Ovarian Cancer

### miRNA in Ovarian Cancer Diagnosis

The utilization of miRNA, as a potential new biomarker, to achieve early diagnosis of OC is of particular importance. A study investigated 31 patients with stage I high-grade serous ovarian cancer who underwent miRNA sequencing and verified the sequencing results in multiple independent data sets. The results showed that the selected miRNAs had a high diagnostic accuracy (AUC = 0.99) in patients with stage I high-grade serous ovarian cancer that outperformed CA125 [38]. One of the approaches is to build an analytic model using computer algorithms. Based on the sequencing results of serum samples and subsequent neural network analysis, an algorithm using miRNA for the diagnosis of epithelial OC was generated (AUC 0.90; 95% confidence interval: 0.81–0.99). Compared with CA125, a traditional marker for OC, the novel model was effective for patients of different ages, tissue types and disease stages, which not affected by the adjustment of the original data. It also showed higher sensitivity in borderline tumors and lower false positive rate. After verification, the positive predictive value of the model was 91.3% (95% CI: 73.3–97.6%) and the negative predictive value was 78.6% (95% CI: 64.2–88.2%). These results demonstrated the diagnostic value of miRNAs and indicated that such algorithm models are worthy of further investigations and optimizations [39]. In basic researches, miRNAs that can distinguish benign and malignant ovarian tumors can be enriched and an expression profile can be constructed [33]. To determine which miRNAs are the most valuable to be included in the algorithm model among all valid miRNAs that have been reported, Cui et al. [40] systematically reviewed 22 studies with a total of 2667 subjects and 8 single miRNAs and found that miR-200c, miR-200a and miR-200b were potential biomarkers with diagnostic value for OC. In summary, the specific expression pattern of miRNAs made them valuable biomarkers for the diagnosis of OC. Constructing algorithm models of miRNAs screened in the map will effectively improve the detection rate of OC, which will also be our future research direction. The advantages and disadvantages of miRNA in different studies are shown in Table 2.

**Table 2:** Diagnostic value of different models

miRNAs	AUC	Sensitivity	Specificity	Diagnostic index	References
miR-320a, miR-665, miR-3184-5p, miR-6717-5p, miR-4459, miR-6076, miR-3195, miR-1275, miR-3185, miR-4640-5p	1.00	100%	100%	98.8%	Yokoi et al. [7]
miR-200b-3p, miR-182-5p	1.00	100%	100%		Záveský et al. [31]
miR-135b-5p	0.980	100%	85.71%		
miR-451a, miR-204- 5p	0.939, 0.878	85.71%	100%		
miR-101-3p, miR-142-5p, miR-148a-3p	0.65				Wiczling et al. [41]
miR-200a, miR-200b, miR-200c, miR-141, miR-429, miR-203a, miR-34a, miR-34b	0.845, 0.822, 0.861, 0.825, 0.791, 0.831, 0.655, 0.832	85.71%, 67.86%, 71.43%, 85.71%, 85.71%, 82.14%, 42.86%, 71.43%	78.33%, 90.00%, 86.67%, 80.00%, 68.33%, 80.00%, 83.33%, 75.00%	80.68%, 82.95%, 81.82%, 81.82%, 73.86%, 80.68%, 70.45%, 73.86%	Márton et al. [42]
miR-182, miR-183, miR-96, miR-182, miR-141, miR-15b, miR-130b, miR-135b, miR-1271, miR-574	0.978	97%	92%	96%	Wang et al. [36]
miR-125b, miR-1290, miR-183, miR-200a, miR-200c, miR-429, miR-1246, miR-4532, miR-142, miR-6076	0.85	78%	78%		Zhang et al. [43]

### miRNA in Ovarian Cancer Treatment

**Fig. 2:**
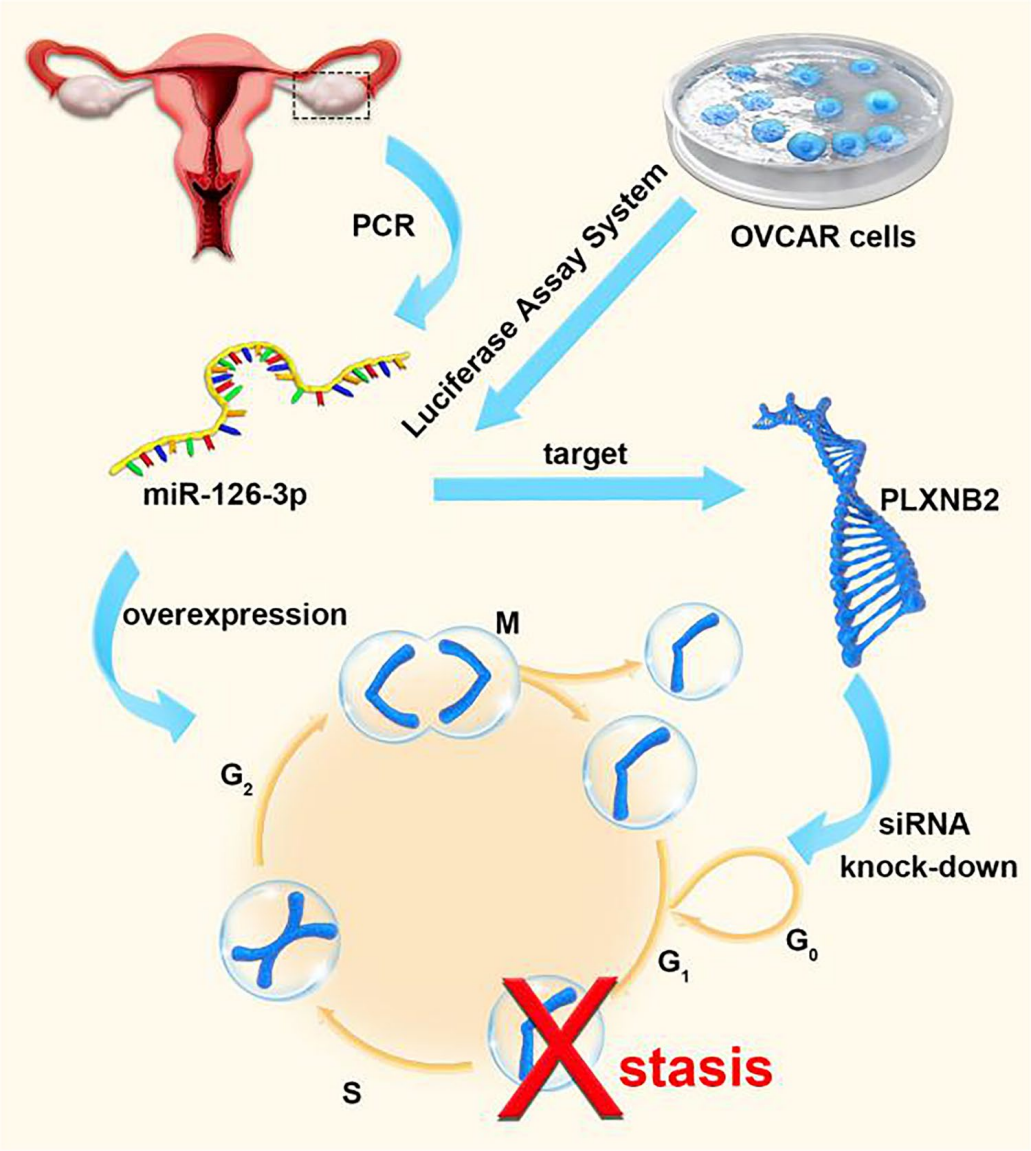
Mechanism of miR-126-3p and PLXNB2 action

Multiple studies using miRNA and cDNA microarrays revealing extensive transcriptional changes in OC have found several miRNAs that are downregulated in advanced or high-grade ovarian cancer, which indicates that miRNAs are involved in malignant tumorigenesis of OCs [44]. Meanwhile, more studies keep discovering new miRNAs that are meaningful in the pathogenesis of ovarian cancer [45–47]. Consistently, these results continue to support the theory that miRNAs participate in the pathogenesis of malignant tumors. Therefore, the possibility to utilize these features of miRNAs to intervene tumor development has become the essential topic of various studies. A set of qRT-PCR data showed that miR-126-3p levels were significantly reduced in OC tissues compared with adjacent normal tissues. Overexpression of miR-126-3p in in vitro experiments induced ovarian cancer cell cycle arrest at the G1/S stage, indicating the inhibiting role of miR-126-3p in proliferation and invasion of OC cells. Further bioinformatics analysis and luciferase assay verification found PLXNB2 to be a functional target of miR-126-3p in OVCAR3 cells. Proliferation and invasion of OC cells were inhibited when PLXNB2 was knocked down by using PLXNB2 siRNA. Similar effect was observed when miR-126-3p was overexpressed in OC cells. Meanwhile, miR-126-3p over-expression and PLXNB2 downregulation were demonstrated to have a synergistic effect on cell growth viability, colony formation and invasion. Therefore, it was believed that miR-126-3p and PLXNB2 can be used as important targets in the treatment of OC [48] (Fig. 2). Typically, tumor metastasis predicts rapid progression of the disease, which is particularly true in OC and poses a treatment challenge. Extensive abdominal metastasis would prevent a radical resection of all cancer foci by surgery, which greatly affects the therapeutic effect and disease prognosis. As a known biomarker for ovarian cancer, miR-200c-3p was found to have a significantly higher expression in advanced and metastatic ovarian cancer. By targeting downregulation of DLC1, it promotes the progression of ovarian cancer [49]. The immune microenvironment, consisting mainly of tumor-associated macrophages and T lymphocytes, plays an important role in the development and metastasis of OC. Studies have pointed out that miR-200b is highly expressed in plasma-derived exosomes from patients with ovarian cancer, and it promotes the proliferation and invasion of ovarian cancer cells by inhibiting the expression of KLF6 to induce the polarization of macrophages M2 [50]. Evidence has shown that the imbalance of T cell subsets in metastatic site is more substantial than that in the primary site. Microarray analysis of macrophage exosomes found that miRNAs enriched in exosomes could regulate the ratio of Treg/Th17 cells, thereby generating an immune microenvironment that promotes the development and metastasis of OC [51]. In addition, cancer cells that spread to the peritoneum normally have limited vascular supply and are in a state of long-term hypoxia, which may aggravate the malignancy and invasiveness of cancer cells. Moreover, some miRNAs can inhibit the progression of OC by downregulating c-Met expression [52]. In summary, miRNAs are important driving factors for the tumorigenesis and development of OC, as well as vital potential targets for preventing the metastasis of cancer cells.

Currently, despite the fact that complete remission of OC can be achieved in up to 80% of patients after standard treatment, half of the them will develop chemotherapy resistance in later period [4]. Studies suggest that drug resistance may be associated with increased expression of drug transporters and extracellular matrix proteins. On the basis of various regulatory effects of miRNAs on cancer, extensive researches have been carried out on miRNAs in ovarian cancer drug resistance [53–55]. X-linked inhibitor of apoptosis (XIAP) is a known factor that causes severe chemoresistance in OC cells. The Cancer Genome Atlas (TCGA) has been used to screen miRNAs with potential survival benefits for patients with OC. One of the regulatory miRNAs, miR-142-5p, has been found to be able to regulate the sensitivity of OC cells to cisplatin by affecting the expression of XIAP [56]. In addition, miR-30-5p was found to promote cell apoptosis and lead to cell cycle arrest at cellular level; while at the gross level, it could significantly reduce the volume of solid tumors [57]. Paclitaxel (PTX) is another first-line chemotherapeutic agent for OC with different resistance mechanism from platinum drugs. Due to the important role of miRNAs in gene expression regulation, researchers tried to find miRNAs against PTX resistance. Consequently, the overexpression of miR-181c was found to be able to inhibit the PI3K/Akt pathway both in vitro and in vivo, which reduced the expression of GRP78 and reversed PTX resistance [58]. For patients with OC who had recurrence due to drug resistance, few treatment options are available. In the face of the substantial cost of targeted therapy, it is essential to explore effective therapeutic targets after drug resistance develops. As several preliminary studies have presented positive results, the optimal therapeutic effect and anti-drug resistance properties of miRNAs have given new insight to future studies in this field.

Latest researches have demonstrated the potential of miRNAs as a new treatment strategy in OC chemotherapy [59]. Firstly, the screening of miRNAs with acquired functions was conducted in combination with real-time continuous cell monitoring to select miRNAs with strong cytotoxicity to drug-resistant OC cells, in which miR-3622b-5p was selected for further research. Results showed that miR-3622b-5p increased the sensitivity of cells to platinum agents, reduced the migration ability and induced the apoptosis of OC cell lines through EGFR. In addition, combined treatment with EGFR inhibitors and small molecule BH3 mimics resulted in a significant reduction of cell viability in both OC patient tissues and OC cell lines. This preliminary study combining miRNA and clinical drug therapy is a major advancement suggesting miRNAs as an optimal therapeutic strategy, which also proves that a new combination therapy based on specific miRNAs is of great value in the treatment of OC.

### miRNA in Ovarian Cancer Prognosis

As a highly malignant disease, OC generally leads to poor prognosis in certain group of patients. Previous studies have demonstrated the close relationship between miRNAs expression and the prognosis of OC. A meta-analysis concluded that miRNAs could affect almost all functions of cancer cells and they could be used as novel prognostic markers since their expression profiles in different tissues as well as the serum and plasma have been fully evaluated [60]. A study found that miR-125b can not only distinguish benign and malignant ovarian tumors, but also has significant differential expression in the serum of patients with different stages of OC, with or without surgical residuals. The results suggest a predictive value of serum miR-125b for the prognosis of patients with OC [61]. Similarly, Brouwer et al. [62] found that the expression of miR-145-5p was related to the disease stage of OC and the expression of miR-145-5p was significantly reduced in advanced-stage patients. When further external verification was performed on a developed prediction model containing 35 miRNAs, results showed an AUC value of 0.68 (95% Cl 0.57–0.79), which fully demonstrated the prognostic value of the miRNAs included in the model [63]. In addition, a later study investigating 197 patients with epithelial OC identified 8 miRNAs with predictive values for overall survival, progression free survival, and chemotherapy resistance. However, further verification using external datasets found that variations among different platforms could affect the results. Therefore, further in-depth studies evaluating the role miRNAs in predicting the survival and recurrence of OC are warranted [64].

## Future Perspectives

As an important driving factor for the progression of OC, the dysregulation of miRNAs plays an important role in the diagnosis, treatment and prognosis of OC. As a hotspot in the field of tumor research, miRNAs showed potential as excellent biological targets for early screening, targeted therapy, drug resistance monitoring and prognosis improvement in malignancies such as OC. However, given its substantial variability and complex biological effects, the specific mechanism of miRNAs in tumors of different tissue types remains largely unclear. Meanwhile, changes in miRNA expression profiles as the tumor progresses have not been clearly identified, with few ongoing studies investigating the predictive ability of miRNAs for OC. Therefore, more in-depth studies are needed to discover new strategies for accurate detection, treatment and prognostic prediction of OC. Moreover, the potential advantage of the combination of mechanistic research and clinical trials should be highlighted to truly enable the value of miRNAs in the treatment of OC.

## Data Availability

Not applicable.
